# Supplementary material for: Avl9 defines a family of GTPase-activating proteins that regulate diverse cell biological functions
Source: bioRxiv. 2026 Jan 5:2026.01.05.697756. Preprint. [Version 1] doi: 10.64898/2026.01.05.697756 (PMC12803157; doi:10.64898/2026.01.05.697756)
Supplement: Supplement 1 [file NIHPP2026.01.05.697756v1-supplement-1.pdf]

bioRxiv preprint doi: https://doi.org/10.64898/2026.01.05.697756; this version posted January 5, 2026. The copyright holder for this preprint (which was not certified by peer review) is the author/funder, who has granted bioRxiv a license to display the preprint in perpetuity. It is made available under aCC-BY 4.0 International license.

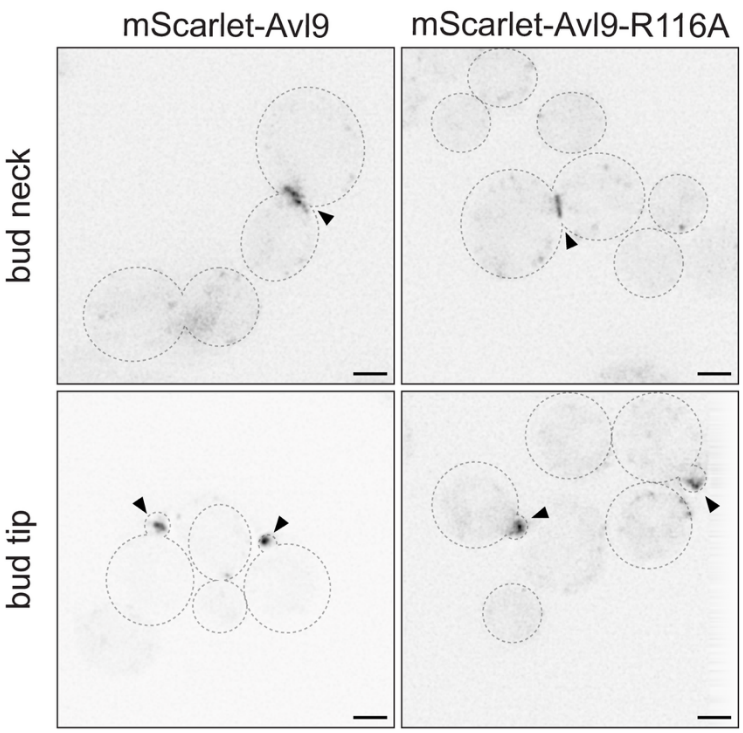

Figure S1. Avl9-R116A expresses and localizes similar to wild-type Avl9.
Live cell fluorescence microscopy images. mScarlet-tagged Avl9 or Avl9-R116A expressed as sole copy of Avl9 from plasmids. Scale bars represent 2 µm.

bioRxiv preprint doi: https://doi.org/10.64898/2026.01.05.697756; this version posted January 5, 2026. The copyright holder for this preprint (which was not certified by peer review) is the author/funder, who has granted bioRxiv a license to display the preprint in perpetuity. It is made available under aCC-BY 4.0 International license.

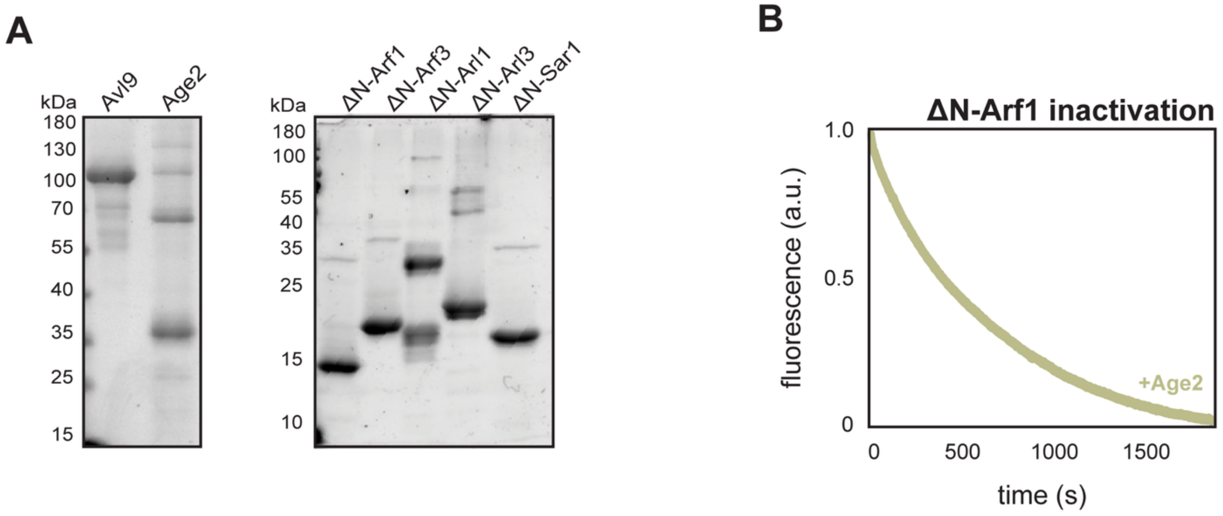

Figure S2. *In vitro* GAP assays.
(**A**) SDS-PAGE gels stained with Coomassie showing ΔN-Arf GTPases, Avl9, and Age2 used in biochemical assays. Note that ΔN-Arl1 appears to form SDS-resistant dimers (~35 kDa) that arise even after isolation of monomeric ΔN-Arl1 fraction via size exclusion chromatography. (**B**) Full reaction span of native tryptophan fluorescence Age2 GAP assay with ΔN-Arf1 (a.u. = arbitrary units).

bioRxiv preprint doi: https://doi.org/10.64898/2026.01.05.697756; this version posted January 5, 2026. The copyright holder for this preprint (which was not certified by peer review) is the author/funder, who has granted bioRxiv a license to display the preprint in perpetuity. It is made available under aCC-BY 4.0 International license.

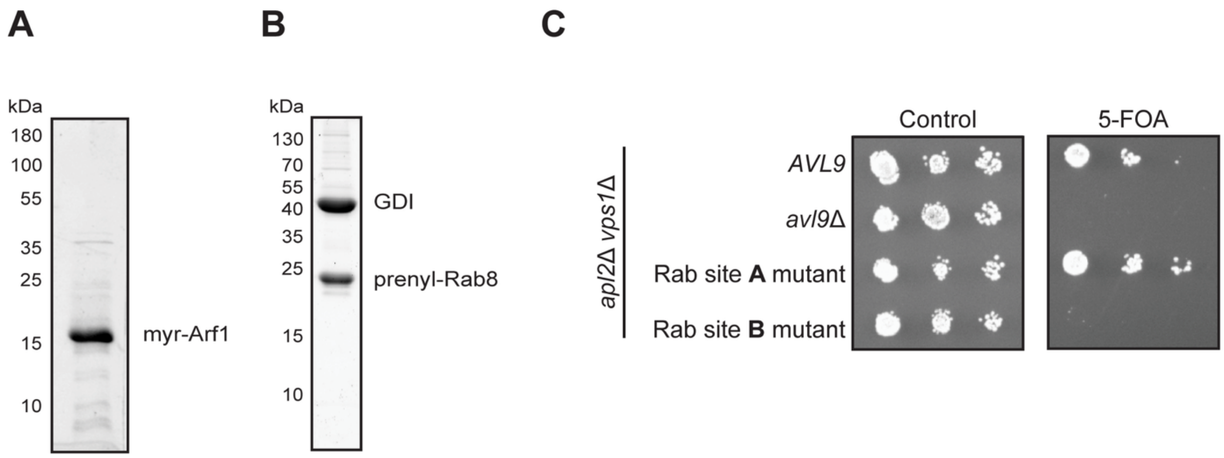

Figure S3. Avl9 is a Rab8 effector.
(**A, B**) SDS-PAGE gels stained with Coomassie showing purified full-length, myristoylated Arf1 and prenylated Rab8-GDI complex. (**C**) Yeast growth assay, cultures were serially diluted from left to right. *URA3* plasmid encoding *APL2* counter-selected on 5-FOA. *AVL9* Rab site A mutant = F261D F272D L461D and Rab site B mutant = I141D G185D D370K.

bioRxiv preprint doi: https://doi.org/10.64898/2026.01.05.697756; this version posted January 5, 2026. The copyright holder for this preprint (which was not certified by peer review) is the author/funder, who has granted bioRxiv a license to display the preprint in perpetuity. It is made available under aCC-BY 4.0 International license.

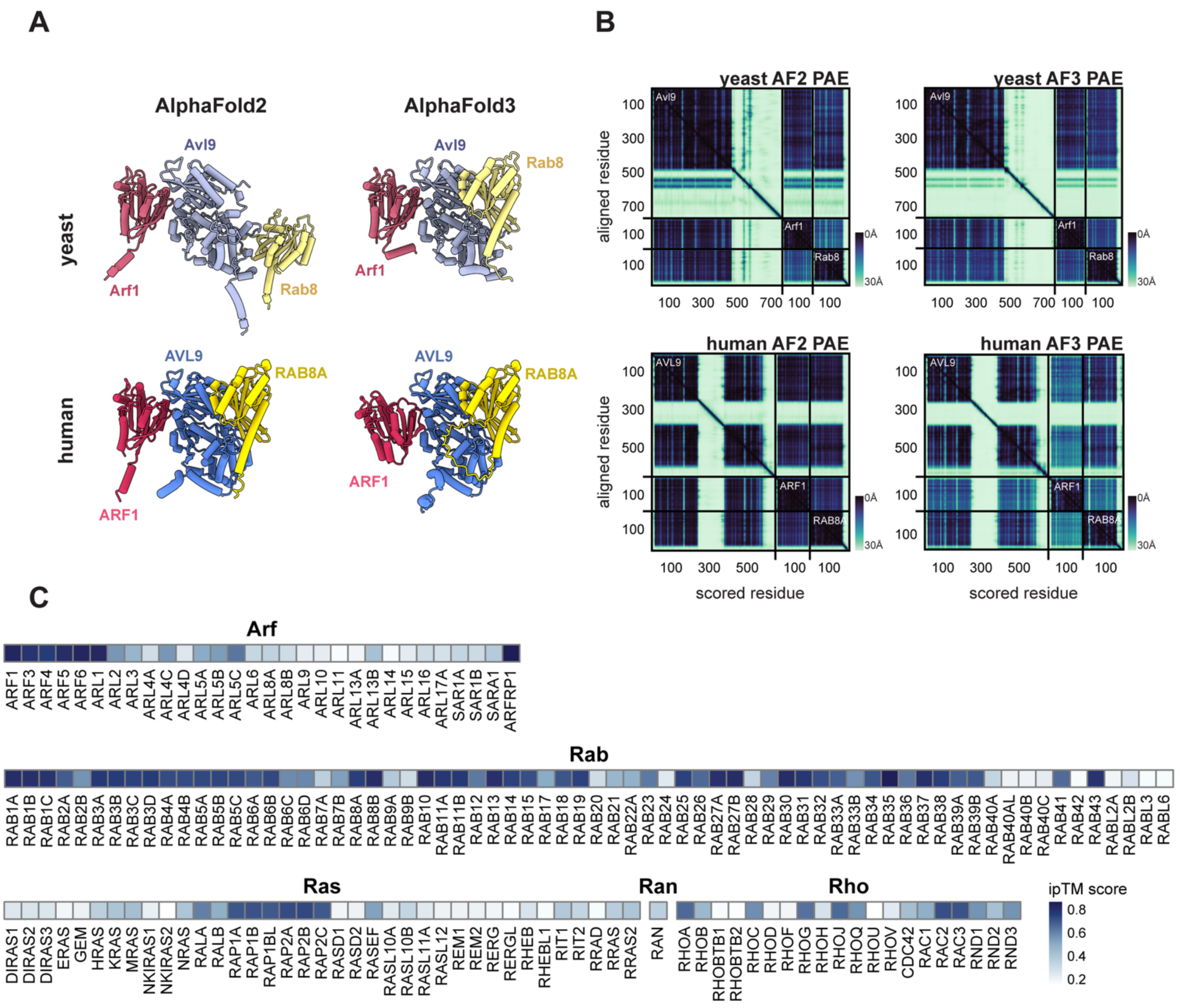

Figure S4. Comparison of yeast and human Avl9-Arf1-Rab8 structural predictions.
(**A**) AlphaFold2 (AF2) and AlphaFold3 (AF3) structural predictions of yeast or human Avl9-Arf1-Rab8 in complex and (**B**) corresponding PAE plots. (**C**) Heatmap showing the average ipTM score of structural predictions for AVL9 and a given GTPase.

bioRxiv preprint doi: https://doi.org/10.64898/2026.01.05.697756; this version posted January 5, 2026. The copyright holder for this preprint (which was not certified by peer review) is the author/funder, who has granted bioRxiv a license to display the preprint in perpetuity. It is made available under aCC-BY 4.0 International license.

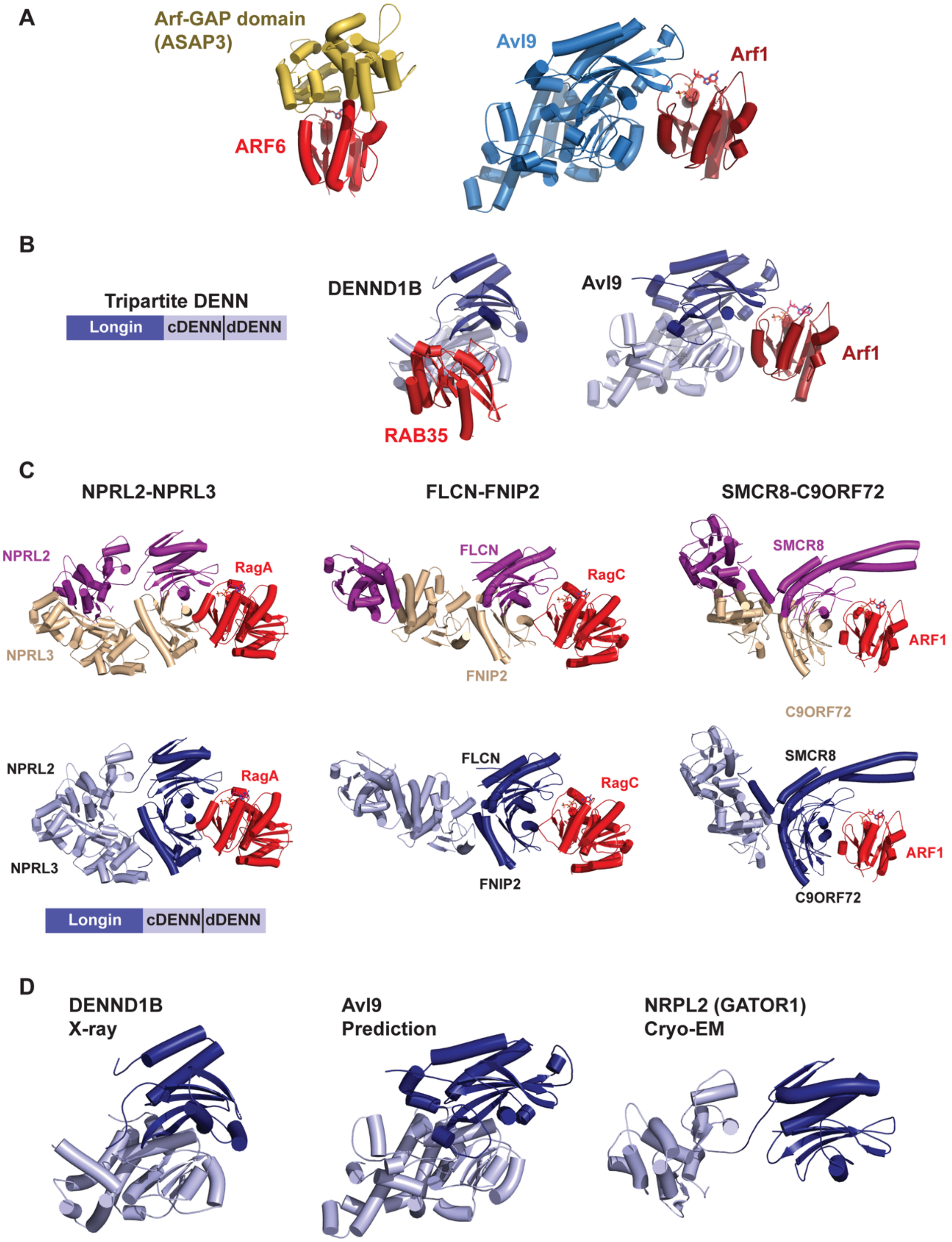

Figure S5. Comparison of DENN domain protein structures.
(**A**) ASAP3-ARF6 X-ray crystal structure[56] and Avl9-Arf1 structural prediction. (**B**) *Left,* schematic denoting the subregions of the tripartite DENN domain. *Right,* comparison of the crystal structure of the Rab-GEF DENND1B interacting with Rab35[58] to the predicted structure of the Avl9-Arf1 interaction. Colors illustrate the subdomain structure. (**C**) Cryo-EM structures of the catalytic subunits of the longin domain GAPs bound to their substrate GTPases, colored by subunit[60,62,64]. (**D**) Comparison of DENN1B, Avl9 prediction, and NRPL2 structures colored by subdomain to illustrate the distinct configuration of the longin domain GAP subunits.

bioRxiv preprint doi: https://doi.org/10.64898/2026.01.05.697756; this version posted January 5, 2026. The copyright holder for this preprint (which was not certified by peer review) is the author/funder, who has granted bioRxiv a license to display the preprint in perpetuity. It is made available under aCC-BY 4.0 International license.

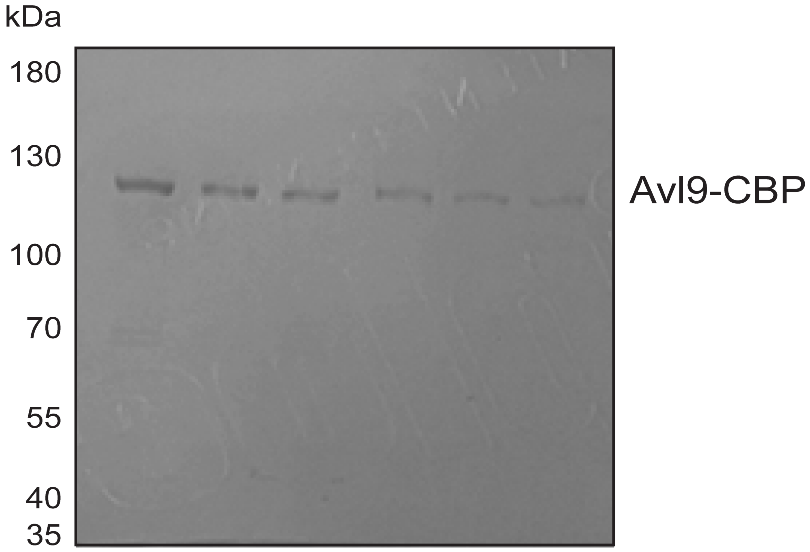

Figure S6. Purification of native Avl9.
SDS-PAGE gel stained with Coomassie. Shown are elution fractions from purification of native *S. cerevisiae* Avl9 with fused calmodulin binding peptide (CBP).

bioRxiv preprint doi: https://doi.org/10.64898/2026.01.05.697756; this version posted January 5, 2026. The copyright holder for this preprint (which was not certified by peer review) is the author/funder, who has granted bioRxiv a license to display the preprint in perpetuity. It is made available under aCC-BY 4.0 International license.

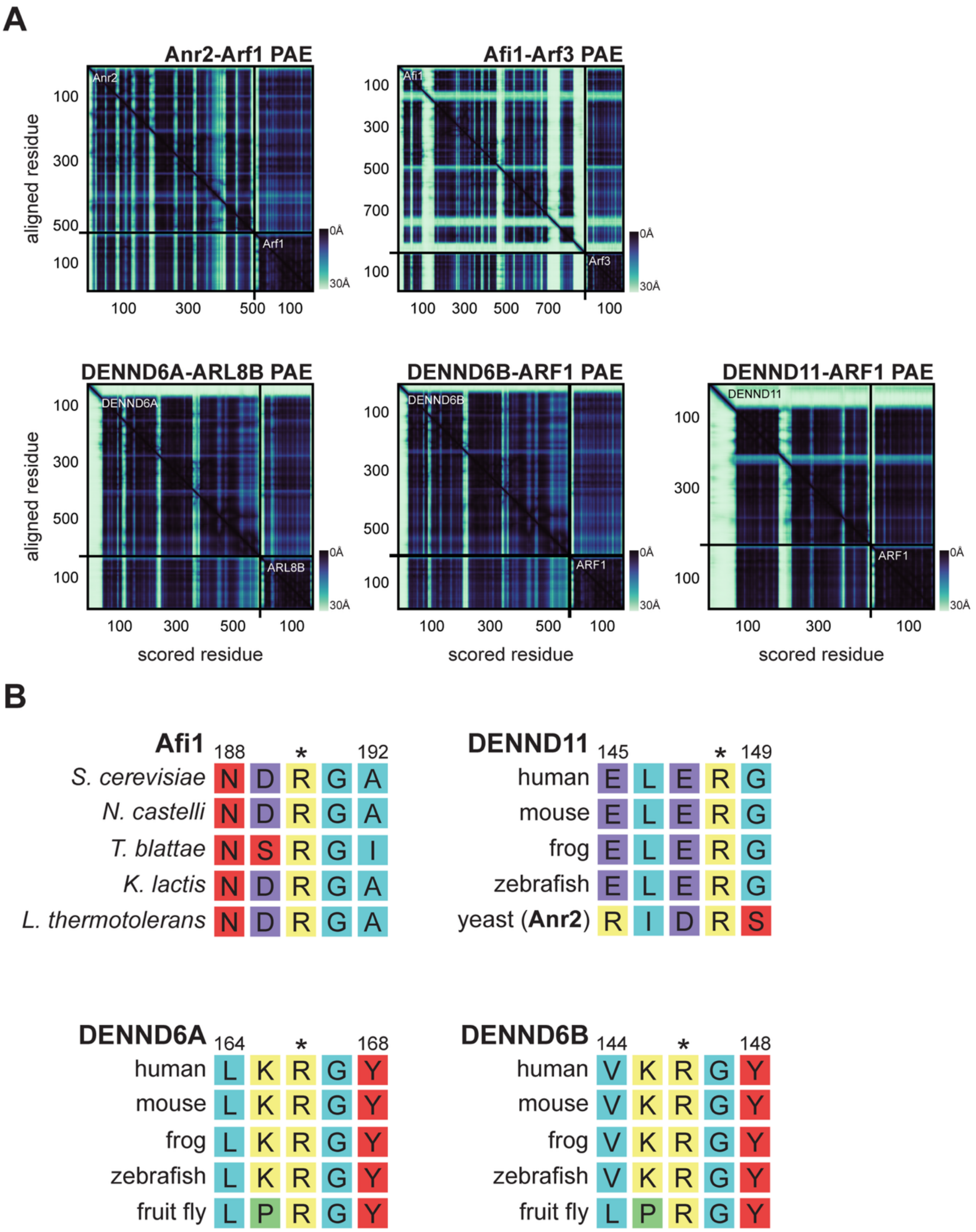

Figure S7. Sequence conservation of predicted arginine finger shared between candidate DENN GAPs.
(**A**) PAE plots of candidate DENN GAP-GTPase predictions (**B**) Multiple sequence alignments of candidate DENN GAP proteins with their homologs in other species. Numbers correspond to amino acids of topmost protein. Predicted catalytic arginine, based on structural predictions, shown with asterisk. For context, levels of genome sequence divergence between *S. cerevisiae* and *K. lactis* is similar to that between humans and starfish[97].

bioRxiv preprint doi: https://doi.org/10.64898/2026.01.05.697756; this version posted January 5, 2026. The copyright holder for this preprint (which was not certified by peer review) is the author/funder, who has granted bioRxiv a license to display the preprint in perpetuity. It is made available under aCC-BY 4.0 International license.

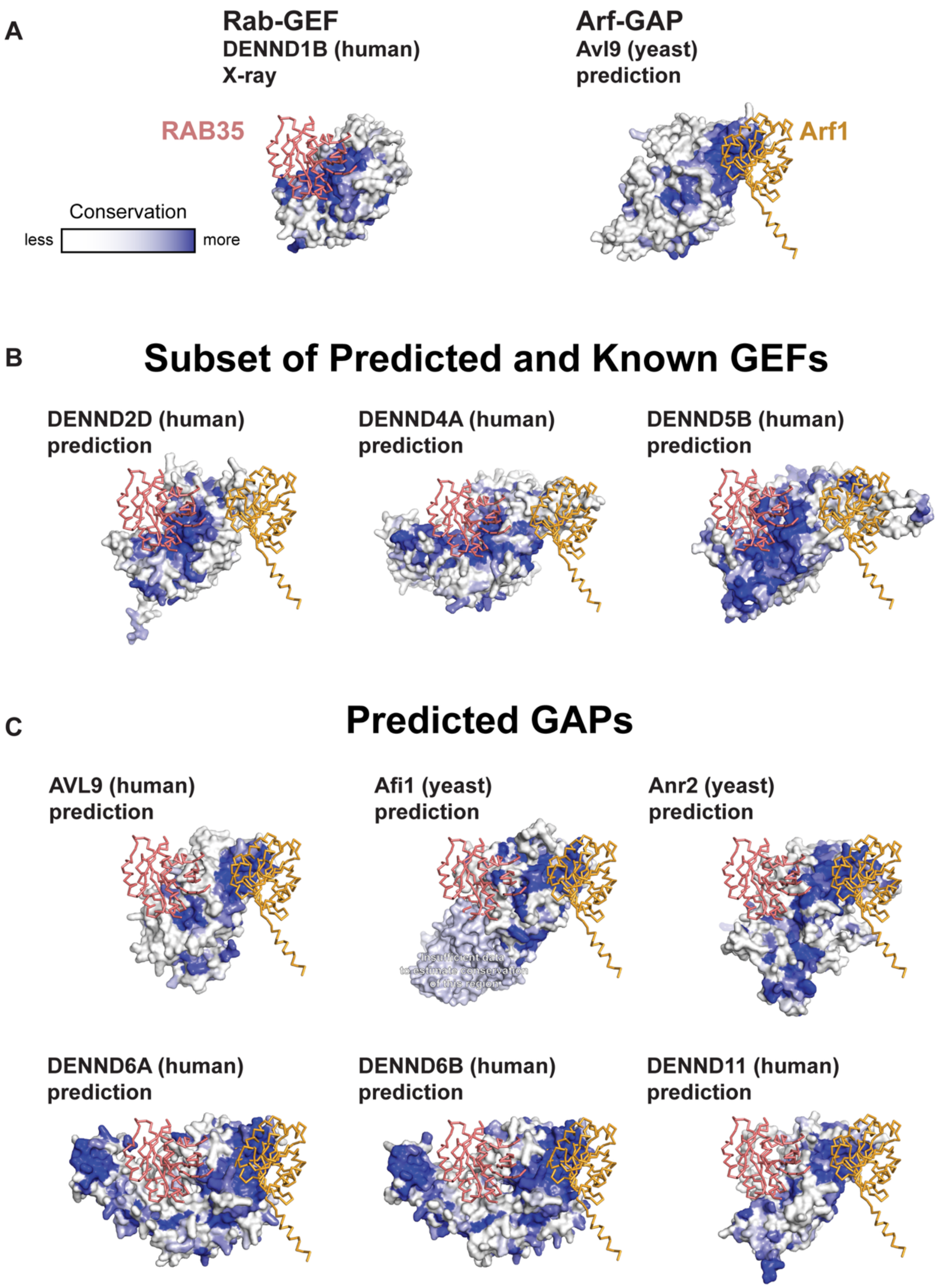

Figure S8. Sequence conservation of “Arf-GAP surface” shared between candidate DENN GAPs. ConSurf analyses of predicted DENN domain structures[98]. (**A**) Rab35 and Arf1 overlaid on each structure based on alignment to known Rab-GEF (DENND1B)[58] and Arf-GAP (Avl9). (**B, C**) Higher degree of sequence conservation at either Rab35 or Arf1 binding sites indicates likelihood of GEF or GAP function of a given DENN domain protein.

bioRxiv preprint doi: https://doi.org/10.64898/2026.01.05.697756; this version posted January 5, 2026. The copyright holder for this preprint (which was not certified by peer review) is the author/funder, who has granted bioRxiv a license to display the preprint in perpetuity. It is made available under aCC-BY 4.0 International license.

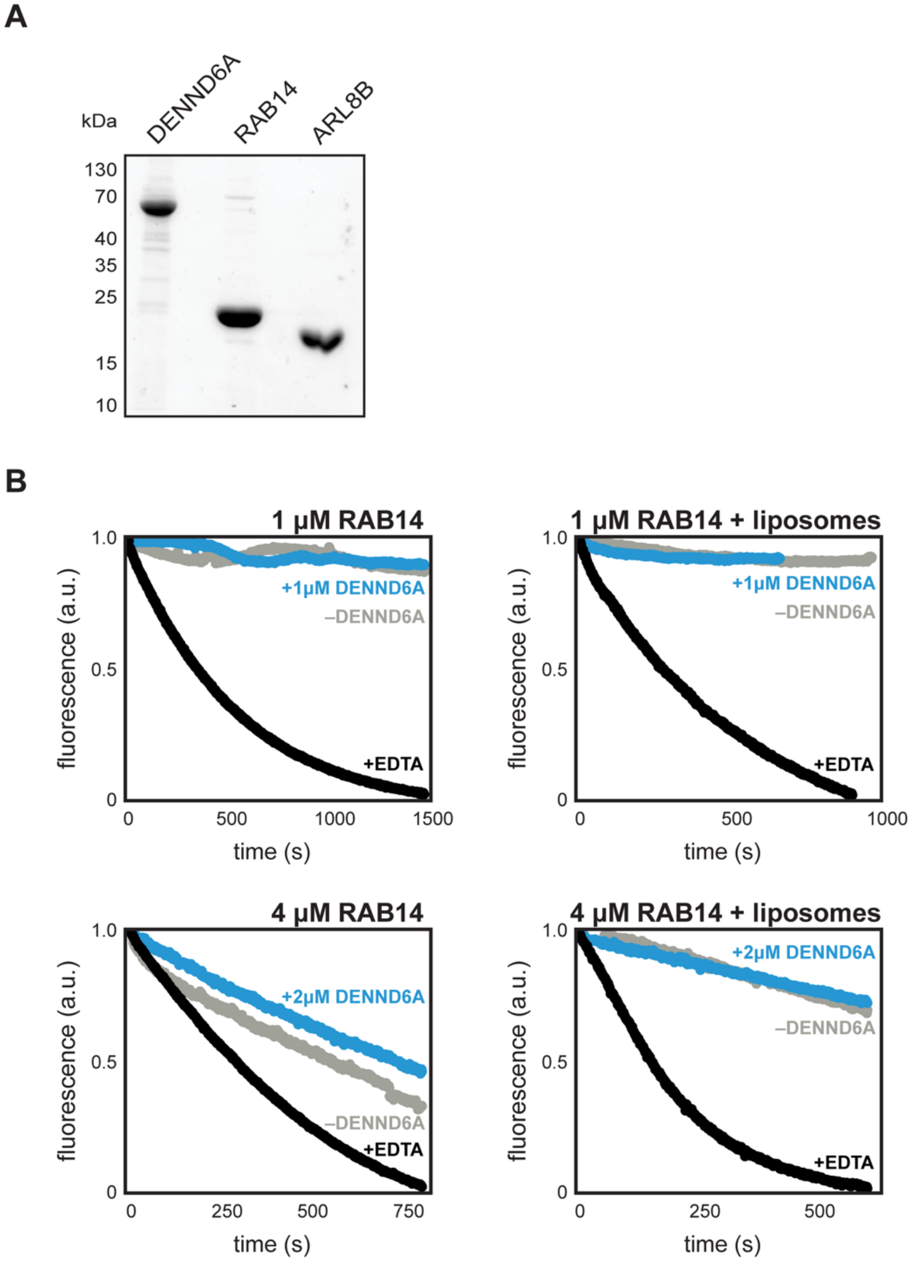

Figure S9. DENND6A *in vitro* GEF assays.
(**A**) SDS-PAGE gel stained with Coomassie showing purified DENND6A, mantGDP-RAB14, and ΔN-ARL8B. (**B**) mantGDP fluorescence GEF assay with RAB14-His. Shown are traces for reactions with DENND6A (blue), without DENND6A (mock; gray) and EDTA (positive control; black).

bioRxiv preprint doi: https://doi.org/10.64898/2026.01.05.697756; this version posted January 5, 2026. The copyright holder for this preprint (which was not certified by peer review) is the author/funder, who has granted bioRxiv a license to display the preprint in perpetuity. It is made available under aCC-BY 4.0 International license.

Table S1. Yeast strains used in this study.

**Name**	**Genotype**	**Source**
SEY6210	*MATα ura3-52 his3-Δ200 leu2-3,112 lys2-801 trp1-Δ901 suc2-Δ9*	Ref. 99
BY4741	*MAT***a** *his3-Δ1 leu2-Δ0 met15-Δ0 ura3-Δ0*	Ref. 100
W303	*MAT***a** *leu2-3,112 trp1-1 can1-100 ura3-1 ade2-1 his3-11,15*	Ref. 101
CFY4946	SEY6210 *avl9*Δ::KanMX	This study
CFY5184	SEY6210 *apl2*Δ::NatMX *vps1*Δ::KanMX *avl9*Δ::His3MX pRS416-*APL2*	This study
CFY5186	SEY6210 mScarlet-*AVL9*::*TRP1*	This study
CFY5234	SEY6210 mScarlet-*avl9*-R111A::*TRP1*	This study
CFY548	W303 *sec7-1*	Ref. 102
CFY5190	W303 *sec7-1 avl9*Δ	This study
CFY5191	W303 *avl9*Δ	This study
KM71H	*aox1*::*ARG4*,*arg4*	Thermo
CFY5269	KM71H *aox1*::*GST-AVL9*	This study
CFY5527	KM71H *aox1*::*GST-DENND6A*	This study
CFY4942	BY4741 *AVL9*-TAP::His3MX	Horizon

bioRxiv preprint doi: https://doi.org/10.64898/2026.01.05.697756; this version posted January 5, 2026. The copyright holder for this preprint (which was not certified by peer review) is the author/funder, who has granted bioRxiv a license to display the preprint in perpetuity. It is made available under aCC-BY 4.0 International license.

Table S2. Plasmids used in this study.

Name	Details	Source
pRS414	Centromeric *TRP1* plasmid	Ref. 103
pRS415	Centromeric *LEU2* plasmid	Ref. 103
pRS416	Centromeric *URA3* plasmid	Ref. 103
CFB4928	pRS414 with Avl9	This study
CFB4936	pRS414 with mScarlet-Avl9	This study
CFB4994	pRS414 with Avl9-(F261D F272D L461D)	This study
CFB5119	pRS414 with mScarlet-Avl9-(F261D F272D L461D)	This study
CFB5372	pRS414 with Avl9-(I141D G185D D370K)	This study
CFB5411	pRS414 with mScarlet-Avl9-(I141D G185D D370K)	This study
CFB5180	pRS414 with Avl9-R116A	This study
CFB5178	pRS414 with mScarlet-Avl9-R116A	This study
CFB5183	pRS416 with Apl2	This study
CFB480	ΔN17-Arf1 with N' TEV-cleavable 6xHis tag	Ref. 90
CFB5272	ΔN16-Arf3 with N' TEV-cleavable 6xHis tag	This study
CFB1435	ΔN13-Arl1 with N' TEV-cleavable 6xHis tag	Ref. 104
CFB1082	ΔN17-Arl3 with N' TEV-cleavable 6xHis tag	This study
CFB3440	ΔN23-Sar1 with N' TEV-cleavable 6xHis tag	Ref. 105
CFB466	pRS415 with GFP-Rab8 (Sec4)	Ref. 106
CFB3545	pRS415 with GFP-Rab11 (Ypt31)	Ref. 73
CFB1018	Rab8-7xHis (Sec4) with N' PreScission-cleavable GST tag	T. Bretscher
CFB2201	Rab11-7xHis (Ypt31) with N' PreScission-cleavable GST tag	Ref. 107
CFB2212	Rab5-7xHis (Vps21) with N' PreScission-cleavable GST tag	Ref. 108
CFB5393	Full-length Rab8 (Sec4) with N' PreScission-cleavable GST tag	This study
pLT40	Gdi1 with N' PreScission-cleavable GST tag	Ref. 73
pLT35	Mrs6 with N' 6xHis tag	Ref. 73
pLT41	Bet2 with N' 6xHis tag and Bet4	Ref. 73
CFB2421	Full-length Arf1 expression plasmid	Ref. 109
CFB2422	Nmt1 expression plasmid	Ref. 109
pcDNA3	Mammalian expression plasmid	Thermo Fisher
CFB5342	pcDNA3 with AVL9	This study
CFB5343	pcDNA3 with AVL9-R111A	This study
CFB5581	lentiCRISPRv2 targeting AVL9: AGATGGCGCACACAACTACC	This study
CFB5523	RAB14-7xHis with N' PreScission-cleavable GST tag	This study
CFB5521	ΔN20-ARL8B with N' TEV-cleavable 6xHis tag	This study

bioRxiv preprint doi: https://doi.org/10.64898/2026.01.05.697756; this version posted January 5, 2026. The copyright holder for this preprint (which was not certified by peer review) is the author/funder, who has granted bioRxiv a license to display the preprint in perpetuity. It is made available under aCC-BY 4.0 International license.

Table S3. Liposome composition.

**Lipid**	**mol %**	
	Golgi	Ni^{2+} Golgi
DOPC	25	20
POPC	6	6
DOPE	7	7
POPE	3	3
DOPS	1	1
POPS	2	2
DOPA	1	1
POPA	2	2
Liver PI	29	29
PI(4)P	1	1
CDP-DAG	2	2
PO-DAG	4	4
DO-DAG	2	2
Ceramide (C18)	5	5
Cholesterol	10	10
Ni^{2+}-DOGS	0	5
